# Supplementary material for: Diet Quality Is Associated with Cardiometabolic Outcomes in Survivors of Childhood Leukemia
Source: Nutrients. 2020 Jul 18;12(7):2137. doi: 10.3390/nu12072137 (PMC7400871; doi:10.3390/nu12072137)
Supplement: Supplementary file 1 [file nutrients-12-02137-s001.pdf]

Supplementary Tables

Supplementary Table S1. Cut-off values and definitions of cardiometabolic outcomes

Risk factor	Adults<br>≥ 18 years old	Children<br>< 18 years old
**Obesity**		
Presenting at least one of two:		
High BMI	≥30 kg/m^2	≥97th percentile
High WC	≥102 cm (men) and ≥88 cm (women)	≥95th percentile
**Insulin resistance**		
Presenting at least one of three:		
High blood fasting glucose	≥6.1 mmol/L (109.8 mg/dL)	≥6.1 mmol/L (109.8 mg/dL)
High glycated hemoglobin	≥6% and <6.5%	≥6% and <6.5%
High HOMA-IR	≥2.86	≥95th percentile of a French-Canadian population
Or		
Taking medication		
**Hypertension**		
Presenting at least one of three:		
Pre-hypertension	≥130/85 and <140/90 mmHg	≥90th and <95th percentile for age and height
Hypertension	≥140/90 mmHg	≥95th percentile for age and height
Or		
Taking medication		
**Dyslipidemia**		
Presenting at least one of three:		
High TG or	≥1.7 mmol/L (150.6 mg/dL)	≥1.47 mmol/L (130.2 mg/dL)
High LDL-C or	≥3.4 mmol/L (131.5 mg/dL)	≥3.36 mmol/L (129.3 mg/dL)
Low HDL-C or	<1.03 (39.8 mg/dL) (men) and <1.3 mmol/L (50.3 mg/dL) (women)	<1.03 mmol/L (39.8 mg/dL)
Or		
Taking medication		

BMI body mass index; HDL-C high-density lipoprotein-cholesterol; HOMA-IR homeostasis model assessment; LDL-C low-density lipoprotein-cholesterol; WC waist circumference, TG triglycerides.

Supplementary Tables

Supplementary Table S2. Associations between adherence to the MEDAS score and anthropometric & metabolic parameters

MEDAS Score							
**Parameters**	**Tertile 1** 2.3 (1–3)[1]	**Tertile 2** 4.0 (3–5)[1]		**Tertile 3** 6.4 (5–10)[1]			
	**OR (95%CI)**	**OR (95%CI)[2]** **Tertile 2 vs. 1**	**P**	**OR (95%CI)[2]** **Tertile 3 vs. 1**	**P**	**OR (95%CI)[2]** **Tertiles 2&3 vs. 1**	**P**
High BMI	-	1.114 (0.38–3.20)	0.84	0.990 (0.32–3.11)	0.99	1.004 (0.39–2.57)	0.99
High WC	-	0.374 (0.14–1.04)	0.06	0.671 (0.25–4.12)	0.44	0.470 (0.19–1.14)	0.09
High SBP	-	1.714 (0.55–5.38)	0.36	1.021 0.25(–4.12)	0.98	1.483 (0.52–4.26)	0.46
High HOMA-IR	-	1.621 (0.59–4.48)	0.35	0.760 (0.23–2.47)	0.65	0.741 (0.30–1.84)	0.50
Low HDL-C	-	0.401 (0.15–1.05)	0.06	0.636 (0.24–1.67)	0.36	0.500 (0.22–1.14)	0.10
High LDL-C	-	1.025 (0.41–2.60)	0.96	0.744 (0.26–2.10)	0.58	1.006 (0.44–2.30)	0.99
High TG	-	1.708 (0.55–5.30)	0.35	0.820 (0.22–3.13)	0.77	1.586 (0.54–4.52)	0.40

[1]Mean score (range); [2]Adjusted for gender, survival time and energy. Cut-off values are described in Supplementary Table 1. BMI, body mass index; WC, waist circumference; BP, blood pressure; HOMA-IR, homeostasis model assessment-insulin resistance; HDL-C, high-density lipoprotein-cholesterol; LDL-C, low density lipoprotein-cholesterol; TG, triglycerides.

Supplementary Tables

Supplementary Table S3. Associations between adherence to the KIDMED score and anthropometric & metabolic parameters

	KIDMED Score						
**Parameters**	**Tertile 1** 0.8 (-2–3)[1]	**Tertile 2** 3.6 (3–4)[1]		**Tertile 3** 5.9 (4–9)[1]			
	**OR (95%CI)**	**OR (95%CI)[2]** **Tertile 2 vs. 1**	**P**	**OR (95%CI)[2]** **Tertile 3 vs. 1**	**P**	**OR (95%CI)[2]** **Tertiles 2&3 vs. 1**	**P**
High BMI	-	1.147 (0.15–8.68)	0.90	1.010 (0.12–8.42)	0.89	1.056 (0.17–6.52)	0.95
High WC	-	0.429 (0.12–1.50)	0.18	0.638 (0.20–2.00)	0.44	0.646 (0.23–1.82)	0.41
High BP	-	0.117 (0.01–1.28)	0.08	0.275 (0.04–1.72)	0.17	0.193 (0.04–1.00)	**0.050**
High HOMA-IR	-	0.675 (0.14–3.37)	0.63	0.629 (0.12–3.28)	0.58	0.615 (0.15–2.51)	0.50
Low HDL-C	-	0.507 (0.09–2.89)	0.45	1.398 (0.31–6.35)	0.67	0.883 (0.22–3.53)	0.86
High LDL-C	-	0.379 (0.05–2.93)	0.35	0.571 (0.08–4.02)	0.57	0.448 (0.08–2.41)	0.35
High TG	-	0.628 (0.09–4.28)	0.64	0.701 (0.13–3.89)	0.68	1.150 (0.23–5.82)	0.87

[1]Mean score (range); [2]Adjusted for gender, survival time and energy. Cut-off values are described in Supplementary Table 1. BMI, body mass index; WC, waist circumference; BP, blood pressure; HOMA-IR, homeostasis model assessment-insulin resistance; HDL-C, high-density lipoprotein-cholesterol; LDL-C, low density lipoprotein-cholesterol; TG, triglycerides.

Supplementary Tables

Supplementary Table S4. Associations between adherence to the HDI-2018 score and anthropometric & metabolic parameters

	HDI-2018 Score						
**Parameters**	**Tertile 1** 2.4 (0–3)[1]	**Tertile 2** 4.0 (3–5)[1]		**Tertile 3** 5.8 (5–9)[1]			
	**OR (95%CI)**	**OR (95%CI)[2]** **Tertile 2 vs. 1**	**P**	**OR (95%CI)[2]** **Tertile 3 vs. 1**	**P**	**OR (95%CI)[2]** **Tertiles 2&3 vs. 1**	**P**
High BMI	-	1.259 (0.49–3.25)	0.63	0.811 (0.30–2.21)	0.63	0.949 (0.41–2.20)	0.90
High WC	-	0.983 (0.48–2.02)	0.96	1.161 (0.57–2.35)	0.68	1.002 (0.54–1.85)	1.00
High BP	-	0.945 (0.37–2.42)	0.91	0.425 (0.14–1.31)	0.14	0.589 (0.25–1.37)	0.22
High HOMA-IR	-	0.894 (0.35–2.26)	0.81	1.302 (0.55–3.11)	0.55	1.109 (0.51–2.41)	0.79
Low HDL-C	-	1.567 (0.73–3.38)	0.25	0.832 (0.37–1.89)	0.66	1.244 (0.63–2.47)	0.53
High LDL-C	-	1.087 (0.46–2.60)	0.85	0.726 (0.29–1.81)	0.49	0.749 (0.35–1.61)	0.46
High TG	-	1.198 (0.47–3.03)	0.70	0.607 (0.21–1.73)	0.35	0.890 (0.39–2.05)	0.78

[1]Mean score (range); [2]Adjusted for gender, survival time and energy. Cut-off values are described in Supplementary Table 1. BMI, body mass index; WC, waist circumference; BP, blood pressure; HOMA-IR, homeostasis model assessment-insulin resistance; HDL-C, high-density lipoprotein-cholesterol; LDL-C, low density lipoprotein-cholesterol; TG, triglycerides.

Supplementary Tables

Supplementary Table S5. Associations between adherence to the HEI-2015 score and anthropometric & metabolic parameters

	HEI-2015 Score							
**Parameters**	**Tertile 1** 47.9 (35–55)[1]	**Tertile 2** 60.0 (55–65)[1]		**Tertile 3** 71.3 (65–85)[1]				
	**OR (95%CI)**	**OR (95%CI)[2]** **Tertile 2 vs. 1**	**P**	**OR (95%CI)[2]** **Tertile 3 vs. 1**	**P**	**OR (95%CI)[2]** **Tertiles 2&3 vs. 1**	**P**
High BMI	-	1.225 (0.46–3.24)	0.68	1.162 (0.44–3.10)	0.76	1.295 (0.55–3.06)	0.56
High WC	-	0.859 (0.43–1.74)	0.67	0.770 (0.38–1.57)	0.47	0.774 (0.41–1.42)	0.41
High BP	-	0.500 (0.18–1.42)	0.19	0.821 (0.32–2.10)	0.68	0.696 (0.31–1.57)	0.38
High HOMA-IR	-	1.660 (0.69–3.98)	0.26	0.947 (0.37–2.44)	0.91	1.207 (0.55–2.64)	0.64
Low HDL-C	-	1.170 (0.56–2.45)	0.68	0.689 (0.31–1.53)	0.36	0.911 (0.47–1.76)	0.78
High LDL-C	-	0.729 (0.31–1.72)	0.47	0.705 (0.29–1.69)	0.43	0.675 (0.32–1.41)	0.30
High TG	-	0.705 (0.28–1.79)	0.46	0.459 (0.17–1.23)	0.14	0.644 (0.28–1.46)	0.29

[1]Mean score (range); [2]Adjusted for gender and survival time. Cut-off values are described in Supplementary Table 1. BMI, body mass index; WC, waist circumference; BP, blood pressure; HOMA-IR, homeostasis model assessment-insulin resistance; HDL-C, high-density lipoprotein-cholesterol; LDL-C, low density lipoprotein-cholesterol; TG, triglycerides.

Supplementary Tables

Supplementary Table S6. Associations between adherence to the FRAP score and anthropometric & metabolic parameters

FRAP Score							
**Parameters**	**Tertile 1** 5.7 (1.96–7.87)[1]	**Tertile 2** 9.8 (7.9–12.4)[1]		**Tertile 3** 20.1 (12.5–48.8)[1]			
	**OR (95%CI)**	**OR (95%CI)[2]** **Tertile 2 vs. 1**	**P**	**OR (95%CI)[2]** **Tertile 3 vs. 1**	**P**	**OR (95%CI)[2]** **Tertiles 2&3 vs. 1**	**P**
High BMI	-	1.372 (0.52–3.59)	0.51	0.556 (0.19–1.67)	0.52	0.946 (0.39–2.33)	0.90
High WC	-	1.259 (0.62–2.56)	0.53	0.994 (0.46–2.17)	0.99	1.144 (0.60–2.19)	0.69
High BP	-	0.723 (0.27–1.96)	0.52	0.518 (0.17–1.55)	0.24	0.625 (0.26–1.53)	0.31
High HOMA-IR	-	0.897 (0.38–2.13)	0.81	0-540 (0.20–1.45)	0.22	0.733 (0.33–1.64)	0.45
Low HDL-C	-	0.749 (0.34–1.64)	0.47	0.603 (0.26–1.41)	0.24	0.682 (0.34–1.39)	0.29
High LDL-C	-	1.615 (0.62–4.17)	0.32	1.247 (0.47–3.33)	0.66	1.429 (0.60–3.40)	0.42
High TG	-	2.460 (0.86–7.00)	0.09	1.870 (0.59–5.95)	0.29	2.217 (0.82–5.99)	0.12

[1]Mean score (range); [2]Adjusted for gender, survival time and energy. Cut-off values are described in Supplementary Table 1. BMI, body mass index; WC, waist circumference; BP, blood pressure; HOMA-IR, homeostasis model assessment-insulin resistance; HDL-C, high-density lipoprotein-cholesterol; LDL-C, low density lipoprotein-cholesterol; TG, triglycerides.

Supplementary Tables

Supplementary Table S7. Association between adherence to nutritional scores and insulin resistance

	Insulin resistance						
**Scores**	**OR (95%CI)**	**OR (95%CI)**	**P**	**OR (95%CI)**	**P**	**OR (95%CI)**	**P**
	**Tertile 1**	**Tertile 2 vs. 1**		**Tertile 3 vs. 1**		**Tertiles 2 & 3 vs. 1**	
MEDAS[2]	-	1.641 (0.59–4.55)	0.34	1.040 (0.34–3.21)	0.95	0.845 (0.35–2.07)	0.71
KIDMED[2]	-	0.675 (0.14–3.37)	0.63	0.629 (0.12–3.28)	0.58	0.615 (0.15–2.51)	0.50
HDI-2018[2]	-	0.903 (0.36–2.28)	0.83	1.512 (0.64–3.56)	0.34	1.212 (0.56–2.61)	0.62
HEI-2015[3]	-	1.746 (0.73–4.16)	0.21	1.017 (0.40–2.58)	0.97	1.284 (0.59–2.79)	0.53
E-DII[3]	-	2.144 (0.93–4.95)	0.07	1.095 (0.42–2.87)	0.85	1.650 (0.75–3.62))	0.21
FRAP[2]	-	0.917 (0.39–2.17)	0.84	0.695 (0.27–1.80)	0.45	0.817 (0.37–1.81)	0.62
% UPF[2,4]	-	0.508 (0.19–1.39)	0.19	0.800 (0.31–2.06)	0.64	0.642 (0.28–1.46)	0.29

Insulin resistance was defined as having at least one of: blood fasting glucose ≥6.1 mmol/L (109.8 mg/dL), glycated hemoglobin ≥6% and <6.5% and homeostasis model assessment-insulin resistance ≥2.86 in adults and ≥95th percentile in children; [2]Adjusted for gender, survival time and energy intake; [3]Adjusted for gender and survival time; [4]%UPF, percentage of contribution of ultra-processed foods to total dietary energy intake using NOVA classification.

Supplementary Tables

Supplementary Table S8. Association between adherence to nutritional scores and dyslipidemia

	Dyslipidemia						
**Scores**	**OR (95%CI) Tertile 1**	**OR (95%CI) Tertile 2 vs. 1**	**P**	**OR (95%CI) Tertile 3 vs. 1**	**P**	**OR (95%CI) Tertiles 2 & 3 vs. 1**	**P**
MEDAS[2]	-	0.584 (0.26–1.31)	0.19	0.603 (0.25–1.44)	0.26	0.653 (0.32–1.33)	0.24
KIDMED[2]	-	0.652 (0.18–2.37)	0.52	1.076 (0.33–3.55)	0.90	1.107 (0.37–3.31)	0.86
HDI-2018[2]	-	1.356 (0.69–2.65)	0.37	0.804 (0.41–1.59)	0.53	1.077 (0.62–1.89)	0.80
HEI-2015[3]	-	0.973 (0.50–1.88)	0.93	0.728 (0.37–1.42)	0.35	0.817 (0.46–1.44)	0.49
E-DII[3]	-	1.445 (0.75–2.80)	0.28	1.572 (0.79–3.13)	0.20	1.502 (0.83–2.71)	0.18
FRAP[2]	-	1.406 (0.71–2.78)	0.33	1.406 (0.68–2.90)	0.36	1.406 (0.76–2.61)	0.28
% UPF [2,4]	-	1.089 (0.52–2.30)	0.82	1.983 (0.93–4.21)	0.08	1.456 (0.76–2.79)	0.26

Dyslipidemia was defined as having at least one of: triglycerides ≥ 1.7 mmol/L (150.6 mg/dL) in adults and ≥ 1.47 mmol/L (130.2 mg/dL) in children, LDL-C ≥ 3.4 mmol/L (131.5 mg/dL) in adults and ≥ 3.36mmol/L (129.9 mg/dL) in children, HDL-C <1.03 in men (39.8 mg/dL), and <1.3 mmol/L (50.3 mg/dL) in women and <1.03mmol/L (39.8 mg/dL) in children; [2]Adjusted for gender, survival time and energy intake; [3]Adjusted for gender and survival time. [4]%UPF: percentage of contribution of ultra-processed foods to total dietary energy intake using NOVA classification.

Supplementary Tables

Supplementary Table S9. Association between adherence to nutritional scores and obesity

	Obesity						
**Scores**	**OR (95%CI) Tertile 1**	**OR (95%CI) Tertile 2 vs. 1**	**P**	**OR (95%CI) Tertile 3 vs. 1**	**P**	**OR (95%CI) Tertiles 2 & 3 vs. 1**	**P**
MEDAS[2]	-	0.599 (0.23–1.53)	0.29	0.799 (0.30–2.10)	0.65	0.571 (0.25–1.31)	0.19
KIDMED[2]	-	0.429 (0.12–1.49)	0.19	0.638 (0.20–2.00)	0.44	0.646 (0.23–1.82)	0.41
HDI-2018[2]	-	1.039 (0.51–2.10)	0.92	1.093 (0.54–2.20)	0.80	1.240 (0.70–2.21)	0.46
HEI-2015[3]	-	0.884 (0.44–1.77)	0.73	798 (0.40–1.61)	0.53	0.801 (0.44–1.46)	0.47
E-DII[3]	-	1.137 (0.57–2.27)	0.72	1.686 (0.82–3.46)	0.16	1.356 (0.74–2.50)	0.33
FRAP[2]	-	1.212 (0.60–2.44)	0.59	0.904 (0.42–1.95)	0.80	1.075 (0.57–2.04)	0.83
% UPF [2,4]	-	0.578 (0.26–1.27)	0.17	1.002 (0.46–2.17)	1.00	0.759 (0.39–1.47)	0.41

Obesity was defined as having at least one of: BMI ≥ 30kg/m^2 in adults and ≥ 97th percentile in children, waist circumference ≥ 102cm in men, ≥ 88cm in women and ≥ 95th percentile in children; [2]Adjusted for gender, survival time and energy intake; [3]Adjusted for gender and survival time; [4]%UPF: percentage of contribution of ultra-processed foods to total dietary energy intake using NOVA classification.

Supplementary Tables

Supplementary Table S10. Association between adherence to nutritional scores and hypertension

	Hypertension						
**Scores**	**OR (95%CI)** **Tertile 1**	**OR (95%CI)** **Tertile 2 vs. 1**	**P**	**OR (95%CI)** **Tertile 3 vs. 1**	**P**	**OR (95%CI)** **Tertiles 2 & 3 vs. 1**	**P**
MEDAS[2]	-	1.714 (0.55–5.38)	0.36	1.021 (0.25–4.12)	0.98	1.483 (0.52–4.26)	0.46
KIDMED[2]	-	0.117 (0.01–1.28)	0.08	0.275 (0.04–1.72)	0.17	0.193 (0.04–1.00)	**0.050**
HDI-2018[2]	-	0.945 (0.37–2.42)	0.91	0.425 (0.14–1.31)	0.14	0.447 (0.20–1.00)	**0.051**
HEI-2015[3]	-	0.500 (0.18–1.42)	0.19	0.821 (0.32–2.10)	0.68	0.696 (0.31–1.57)	0.38
E-DII[3]	-	3.029 (1.00–9.11)	**0.049**	1.135 (0.35–3.71)	0.83	1.928 (0.68–5.44)	0.21
FRAP[2]	-	0.723 (0.27–1.96)	0.52	0.518 (0.17–1.55)	0.24	0.609 (0.27–1.39)	0.24
% UPF [2,4]	-	0.781 (0.24–2.57)	0.68	1.078 (0.36–3.24)	0.89	0.934 (0.35–2.53)	0.89

Pre-hypertension and hypertension were defined, respectively, as: blood pressure ≥130/85 and <140/90 mmHg in adults and ≥90th and <95th percentile for age and height in children and ≥140/90 mmHg or taking medication in adults and ≥95th percentile for age and height or taking medication in children; [2]Adjusted for gender, survival and energy intake; [3]Adjusted for gender and survival time; [4]%UPF: % contribution of ultra-processed foods to total dietary energy intake using NOVA classification.

Supplementary Tables

Supplementary Table S11. Association between adherence to nutritional scores and TNF-α levels in all participants

**Scores**	**Mean ± SD**	**Mean ± SD**	**Mean ± SD**	**P**	**P**	**P**
	**Tertile 1**	**Tertile 2**	**Tertile 3**	**Tertile 3 vs.1**	**Tertile 3 vs. 2**	**Tertile 2 vs. 1**
MEDAS	2.07 ± 0. 57	3.12 ± 7.38	2.25 ± 1.12	0.82	0.77	0.46
KIDMED	2.50 ± 1.07	2.04 ± 0.60	2.11 ± 0.55	0.22	0.65	0.12
HDI-2018	2.24 ± 0.78	2.16 ± 0.90	2.76 ± 5.97	0.24	0.98	0.27
HEI-2015	2.28 ± 0.80	2.89 ± 5.99	1.99 ± 0.53	**0.01**	0.06	0.53
E-DII	2.10 ± 0.89	2.16 ± 0.70	2.89 ± 5.93	0.07	0.38	0.32
FRAP	2.16 ± 0.86	2.25 ± 0.76	2.75 ± 5.94	0.76	0.17	0.36
% UPF[1]	2.04 ± 0.72	2.86 ± 6.71	2.30 ± 1.08	0.11	0.15	0.77

[1]%UPC, percentage of contribution of ultra-processed foods to total dietary energy intake using NOVA classification.

Supplementary Tables

Supplementary Table S12. Association between adherence to nutritional scores and adiponectin levels in all participants

Scores	Mean ± SD	Mean ± SD	Mean ± SD	P	P	P
	**Tertile 1**	**Tertile 2**	**Tertile 3**	**Tertile 3 vs. 1**	**Tertile 3 vs. 2**	**Tertile 2 vs. 1**
MEDAS	14.10 ± 6.86	15.81 ± 7.55	19.41 ± 8.65	**0.004**	**0.004**	0.32
KIDMED	16.66 ± 8.98	18.17 ± 8.81	13.47 ± 6.89	0.27	0.06	0.54
HDI-2018	16.40 ± 9.16	14.45 ± 6.88	17.58 ± 7.73	0.31	**0.02**	0.46
HEI-2015	15.92 ± 8.45	16.44 ± 8.94	16.37 ±6.84	0.51	0.62	0.93
E-DII	17.61 ± 7.33	15.57 ± 8.58	15.59 ± 8.05	0.13	0.77	0.06
FRAP	17.45 ± 7.95	15.13 ± 8.12	16.32 ±7.98	0.36	0.26	0.05
% UPC[1]	18.11 ± 9.08	16.61 ± 7.28	14.74 ± 7.32	0.09	0.30	0.55

[1]%UPC, % contribution of ultra-processed foods to total dietary energy intake using NOVA classification.

Supplementary Tables

Supplementary Table S13. Association between adherence to nutritional scores and IL-6 levels in all participants

Scores	Mean ± SD	Mean ± SD	Mean ± SD	P	P	P
	**Tertile 1**	**Tertile 2**	**Tertile 3**	**Tertile 3 vs. 1**	**Tertile 3 vs. 2**	**Tertile 2 vs. 1**
MEDAS	0.70 ± 1.30	0.51 ± 0.58	0.56 ± 0.43	0.994	0.201	0.170
KIDMED	0.49 ± 0.57	0.44 ± 0.51	0.61 ± 0.94	0.820	0.568	0.398
HDI-2018	0.56 ± 0.59	0.72 ± 1.18	0.41 ± 0.33	0.059	0.117	0.865
HEI-2015	0.56 ± 0.59	0.66 ± 1.08	0.47 ± 0.59	0.164	0.184	0.997
E-DII	0.54 ± 0.64	0.57 ± 1.05	0.58 ± 0.65	0.795	0.765	0.994
FRAP	0.63 ± 1.18	0.53 ± 0.42	0.53 ± 0.57	0.148	0.714	0.117
% UPF[1]	0.51 ± 0.46	0.42 ± 0.33	0.79 ± 1.34	0.814	0.187	0.378

[1]%UPC, % contribution of ultra-processed foods to total dietary energy intake using NOVA classification.

Supplementary Tables

Supplementary Table S14. Association between adherence to nutritional scores and IL-6 levels in obese participants

Scores	Mean ± SD	Mean ± SD	Mean ± SD	P	P	P
	**Tertile 1**	**Tertile 2**	**Tertile 3**	**Tertile 3 vs. 1**	**Tertile 3 vs. 2**	**Tertile 2 vs. 1**
MEDAS	1.13 ± 2.07	0.58 ± 0.55	0.52 ± 0.36	0.26	0.92	0.30
KIDMED	0.42 ± 0.27	0.56 ± 0.53	0.41 ± 0.26	0.97	0.83	0.97
HDI-2018	0.58 ± 0.50	0.83 ± 1.71	0.51 ± 0.35	0.64	0.85	0.76
HEI-2015	0.49 ± 0.37	0.96 ± 1.68	0.44 ± 0.29	0.85	0.10	0.21
E-DII	0.60 ± 0.39	0.85 ± 1.75	0.49 ± 0.41	0.32	0.85	0.37
FRAP	0.88 ± 1.85	0.58 ± 0.32	0.50 ± 0.38	0.88	0.27	0.23
% UPF[1]	0.56 ± 0.37	0.65 ± 0.42	0.83 ± 1.93	0.08	**0.04**	0.49

[1]%UPC, % contribution of ultra-processed foods to total dietary energy intake using NOVA classification.

Supplementary Tables

Supplementary Table S15. Association between adherence to nutritional scores and IL-6 levels in non-obese participants

**Scores**	**Mean ± SD**	**Mean ± SD**	**Mean ± SD**	**P**	**P**	**P**
	**Tertile 1**	**Tertile 2**	**Tertile 3**	**Tertile 3 vs. 1**	**Tertile 3 vs. 2**	**Tertile 2 vs. 1**
MEDAS	0.47 ± 0.42	0.49 ± 0.60	0.58 ± 0.47	0.37	0.13	0.35
KIDMED	0.51 ± 0.71	0.39 ± 0.50	0.72 ± 1.15	0.67	0.74	0.35
HDI-2018	0.55 ± 0.63	0.66 ± 0.82	0.35 ± 0.31	**0.02**	**0.05**	0.92
HEI-2015	0.60 ± 0.67	0.50 ± 0.52	0.48 ± 0.70	0.10	0.52	0.31
E-DII	0.52 ± 0.73	0.43 ± 0.33	0.63 ± 0.75	0.29	0.64	0.60
FRAP	0.53 ± 0.75	0.50 ± 0.47	0.55 ± 0.64	0.12	0.53	0.40
% UPF[1]	0.47 ± 0.51	0.34 ± 0.26	0.77 ± 0.93	**0.10**	**0.004**	0.28

[1]%UPC, % contribution of ultra-processed foods to total dietary energy intake using NOVA classification.
